# Book Review: How Learning Happens: Seminal Works in Educational Psychology and What They Mean in Practice

**DOI:** 10.3389/fpsyg.2021.714290

**Published:** 2021-07-14

**Authors:** Xiqin Chi

**Affiliations:** School of Marxism, Tianjin University of Technology and Education, Tianjin, China

**Keywords:** educational psychology, learning, practice, independent convergence, seminal works

“How learning happens” should be an engaging question automatically asked by teachers, educational psychologists, and anyone interested in the workings of the mind. Of the studies that trace the nature of learning, some seminal works offer prominent observations about learning and the elusive way in which true learning happens. Kirschner and Hendrick bring together 28 of these works and their most important discoveries, as independently verified in the wider scientific community. It is an endeavor to provide a roadmap to reach a broad consensus on a set of common, agreed principles.

The book consists of 28 chapters and is clearly divided into six parts. Part 1 contains five chapters spelling out how our brain works and what that means for teaching and learning. Part 2 discusses the prerequisites of effective, efficient, and enjoyable learning, delineating issues including self-regulated learning, self-efficacy, and goal orientation. Part 3 recounts the learning activities that support learning, namely, scaffolding, effective problem-solving, and mathemagenic activities. The six chapters in Part 4 dwell on the role of teachers and give some tips to help them live up to their positive influence potential, involving simple-to-complex elaboration, explicit instructional guidance, and effective feedback. Part 5 elaborates on different social influences on learning, comprising situated cognition, cognitive apprenticeship, and communities of practice. Lastly, Part 6 is devoted to cautionary tales and prevalent myths and fables that actually hinder learning rather than facilitate it.

This unique book is worth reading because it contributes to demonstrations of diverse theoretical perspectives on how learning happens, shifts the understanding of learning away from philosophical speculation to empirical analysis based on testable hypothesis, and offers practical implications for teaching practice. The three reasons that this book is so highly recommendable are as follows. First, it is an analytical book aimed to show readers the most important research publications on learning and their hard-earned discoveries. The works chosen come primarily from the fields of educational psychology and cognitive psychology. Each chapter introduces a trajectory of thought on a particular area that has left a mark on how we teach today, inspires many teachers and researchers, and will lead to more investigation of that area. Moreover, for each publication, the book follows the same structure by first providing a summary of the research, then listing a series of practical suggestions for how the findings might inform teaching practice. It also offers takeaways and references for each article, as well as suggested reading and links. This analytical nature is effective and efficient to make implicit knowledge explicit.

Second, the book favors the principle of independent convergence. Educational psychology is dependable science, and evidence from many directions moves toward a broad consensus of how we learn (Pomerance et al., 2016). However, there is a reflex-like tendency to reject new evidence or knowledge because it contradicts established norms, beliefs, or paradigms, which some scholars have critiqued as rooted in the scientific management and neo-managerial norms that seek to disseminate knowledge in formalized, routine, and procedural ways that lack appropriate grounding in the science of learning (Myran and Sutherland, 2019). For example, this book uncovers the fact that the cognitive architecture of the brain has been ignored in education despite the transformative change it would bring. Additionally, instructional design in pedagogy is largely based on theory drawing from tangential fields, often leaving research on psychology, cognition, and the brain omitted (Kirschner and Hendrick, 2020). The book further reveals the cumulative nature of learning knowledge as a perpetually changing process that is redefined and built upon previous knowledge and discovery.

Third, this book situates its content in a broader scientific context. Each of the book's 28 chapters examines an important and prominent research paper in the field of education and explains its significance before describing the research, its practical implications, how it can be used in the classroom and the key takeaway for teachers. This leaves space for teachers to gain an appropriate understanding of the theories behind their practice and proper measures for their effective teaching. It not only keeps up with the latest findings, but also seeks sincere, authentic dialogue with the seminal works. The information offered alongside each seminal article, with ready links to external resources, does a wonderful job of providing practical guidance on how these well-established findings might be used to inform teaching practice and design and develop the best learning experiences for students.

With all the merits mentioned above, this book could have been more comprehensive if it provided a timeline of the seminal works in educational psychology, as research on learning is recursive in nature, as is standing on the shoulders of giants. Readers may then get a panoramic picture of the most important and prominent research papers in the field of education and benefit more from implementing evidence-informed education. It would also have been more helpful to chronologically understand the progress painstakingly made by ancestors and the cumulative scientific discovery of how learning happens.

Overall, this book is informative, well-organized, and easy to understand. It should be essential reading for teachers wanting to fully engage with and understand educational research, as well as for undergraduate students in the fields of education, educational psychology, and the learning sciences. It is also a valuable reference book for educators interested in the workings of the mind and anyone who wants to understand how research can improve teaching.

## Author Contributions

The author confirms being the sole contributor of this work and has approved it for publication.

## Conflict of Interest

The author declares that the research was conducted in the absence of any commercial or financial relationships that could be construed as a potential conflict of interest.
